# A retrospective analysis of eosinophilia as a predictive marker of response and toxicity to cancer immunotherapy

**DOI:** 10.2144/fsoa-2020-0070

**Published:** 2020-08-03

**Authors:** Tharani Krishnan, Yoko Tomita, Rachel Roberts-Thomson

**Affiliations:** 1 Medical Oncology, Royal Adelaide Hospital, South Australia 5000, Australia; 2 Medical Oncology, The Queen Elizabeth Hospital, South Australia 5011, Australia

**Keywords:** biomarker, cancer, eosinophilia, immune checkpoint inhibitors, response, toxicity

## Abstract

**Aim:**

To investigate eosinophilia as a potential on-treatment biomarker for patients receiving cancer immunotherapy.

**Materials & methods:**

We evaluated the association between eosinophilia and treatment response and toxicity in a retrospective cohort of patients receiving cancer immunotherapy.

**Results:**

The study involved 146 patients. Eosinophilia developed in 22%. Patients who developed eosinophilia were more likely to achieve disease control (p = 0.009), with every 0.1 × 10^9^/l rise in eosinophil count, while receiving treatment was associated with a 28% relative increased chance achieving disease control. Although there was a trend toward improved survival, there was no significant association between eosinophilia and improved overall survival (p = 0.136). Patients with eosinophilia were more likely to develop toxicity (p = 0.042).

**Conclusion:**

Eosinophilia is a potentially useful biomarker warranting further prospective clinical investigation.

## Lay abstract

Immunotherapy has revolutionized the way we treat many cancers. However, we do not have good predictors for which patients are going to respond to treatment or experience side effects. ‘Eosinophilia’ is when a particular type of white blood cell is present at high levels in the blood. This can occur in patients on immunotherapy. This paper reports a possible link between eosinophilia and the likelihood of responding well to immunotherapy but also the possibility of developing side effects.

Immunotherapy with PD-1, PD-L1 and CTLA4 inhibitors have changed the treatment paradigm for patients with cancer. These agents have demonstrated efficacy in multiple cancers [1–4]. PD-1, PD-L1 and CTLA4 are immune checkpoint molecules that downregulate T cell activation, allowing tumor cells to evade the host immune system. Blocking PD-1, PD-L1 or CTLA4 improves T cell function and augments the host immune response, leading to enhanced tumor killing and inhibition of tumor growth [5]. Multiple immune checkpoint inhibitors have been developed and are available in the medical oncology clinic for use to treat a variety of cancers.

However, the benefit of immunotherapy comes at a considerable cost, both in terms of treatment toxicity and financial cost. Immune related adverse events can affect multiple body systems causing varying manifestations including rash, pneumonitis, colitis, thyroid dysfunction, hypophysitis, diabetes, hepatitis, nephritis, myocarditis and neuropathy [6]. Furthermore, a number of these toxicities are irreversible and require lifelong hormone replacement or prolonged immunosuppression. There is also a significant financial burden associated with immune checkpoint inhibitors, with each treatment cycle costing thousands of dollars. This has important economic consequences for healthcare systems worldwide, especially since not all patients will respond to immunotherapy treatment.

Therefore, determining on-treatment biomarkers for response and toxicity has far-reaching implications. Numerous biomarkers have already been proposed, many of which involve the immunologic aspects of the tumor microenvironment [7,8]. Overexpression of PD-L1 by tumor cells or immune cells in the tumor microenvironment is a commonly used biomarker, but some patients respond to treatment even with negative or low PD-L1 expression and a standard method of detection and measurement of PD-L1 status is still being established. Other exploratory biomarkers require complex tumor molecular testing, specific gene tests or serum tests for specific cell types, which are costly, time consuming and have demonstrated varied results for different tumor types. Examples include microsatellite instability, T-cell subset analysis, tumor mutational burden with gene sequencing and tumor-infiltrating lymphocytes [7].

Eosinophil count is a widely available blood parameter and part of the routine complete blood examination performed for patients on immunotherapy. Patients treated with immunotherapy can develop eosinophilia on routine testing and there are multiple possible mechanisms by which eosinophils can affect the tumor environment [9–11]. Studies in melanoma patients have determined that patients who developed eosinophilia on treatment had a significantly prolonged survival, suggesting a prognostic role for eosinophilia. Thus, eosinophilia has emerged as a potential on-treatment biomarker.

The aims of this study were to review the incidence of emerging eosinophilia in patients treated with immune checkpoint inhibitors, and to explore the relationship between the development of eosinophilia and disease outcomes and toxicity in a real-world setting.

## Materials & methods

### Patients

Data were provided from patients treated with immune checkpoint inhibitors at The Queen Elizabeth Hospital (TQEH; Adelaide, South Australia). Inclusion criteria were all patients aged 18 years or older, treated with any immune checkpoint inhibitor for a solid tumor at TQEH and who had received at least one cycle of treatment. This study was approved by the Central Adelaide Local Health Network (South Australia) Human Research Ethics Committee on 26 November 2018 (approval Q20180512).

### Study design

A retrospective case review analysis was undertaken. Eligible patients were identified from the pharmacy database at TQEH. The patients’ data were collected from South Australia's health electronic medical record (Sunrise EMR) at TQEH, and from an alternative program (OACIS) if patients were treated prior to the introduction of Sunrise EMR. The data were collected between January and May 2019. All patient data were de-identified.

Demographic data were collected, including patient age, gender and Eastern Cooperative Oncology Group (ECOG) performance status. Baseline tumor characteristics including tumor type, cancer stage, BRAF mutation status, PD-L1 expression, LDH at baseline, the presence or absence of brain metastases, and previous treatments (systemic treatment, corticosteroids and radiotherapy) were recorded. Immunotherapy treatment details collected were; the start date of treatment; number of cycles received; best radiological response (complete response, partial response, stable disease or progressive disease) and date of progression. Where applicable, the date of death and time from treatment commencement to death were recorded. All immune related adverse events were recorded, with a toxicity grade and whether or not systemic corticosteroids were required. The data were entered and stored on a secure hospital compute requiring password access.

The leukocyte count was recorded at baseline, prior to immunotherapy treatment. The eosinophil count was recorded at baseline, cycle 2 and week 6 of treatment, as well as the peak eosinophil count on treatment. Eosinophilia was defined as a peripheral blood absolute eosinophil count greater than 0.5 × 10^9^/l.

### Statistics

All statistical analysis was performed using IBM SPSS Statistics version 25. Descriptive statistical analysis was performed to examine the baseline characteristics of the included patients. Fisher's exact test was used to assess: the association between treatment response and eosinophilia, and the association between toxicity and eosinophilia. Peak eosinophil count on treatment was used for these analyses. A binary logistic regression analysis was utilized to assess the association between disease control on treatment and a rise in eosinophil count from baseline. A Kaplan–Meier survival analysis with log-rank test was used to assess the association between eosinophilia on treatment and survival – excluding patients who had treatment in the adjuvant setting. Statistical significance of all tests was accepted for values of p < 0.05.

## Results

### Patient characteristics

A total of 146 patients were included in the study. The baseline characteristics of the patients are shown in Table 1. 60% of patients were male (n = 88). The median age was 68 years (range 22–95 years), and the majority had a good performance status (ECOG 0 to 1).

**Table 1.** Baseline characteristics of the included patients.

Descriptor	Number (%)
Total patient cohort	146 patients
Gender – Male – Female	88 (60.2) 58 (39.8)
Median age at diagnosis	62.9 years (range 22–95 years)
ECOG performance status – 0 – 1 – 2 – ≥3 – Unknown	36 (24.7) 41 (28.1) 9 (6.2) 4 (2.7) 56 (38.4)
Stage – II–III – IV	15 (10.3) 131 (89.7)
Brain metastases	18 (12.3)
Raised LDH at immunotherapy commencement	74 (50.7)
Previous radiotherapy	65 (44.5)
Corticosteroids at time of treatment commencement^†^	12 (8.2)
Line of treatment – 1 – 2 – 3 – 4 or more	64 (43.8) 51 (34.9) 17 (11.6) 11 (7.5)

Study patients numbered 146. The mean age was 63 years, 60% were male and most had an ECOG performance status of 0 to 1. The majority of the patients had stage IV disease, and most were receiving immunotherapy as first or second-line treatment.

† (dose equivalent to 10 mg prednisolone per day).

ECOG: Eastern Cooperative Oncology Group.

**Figure 1.**
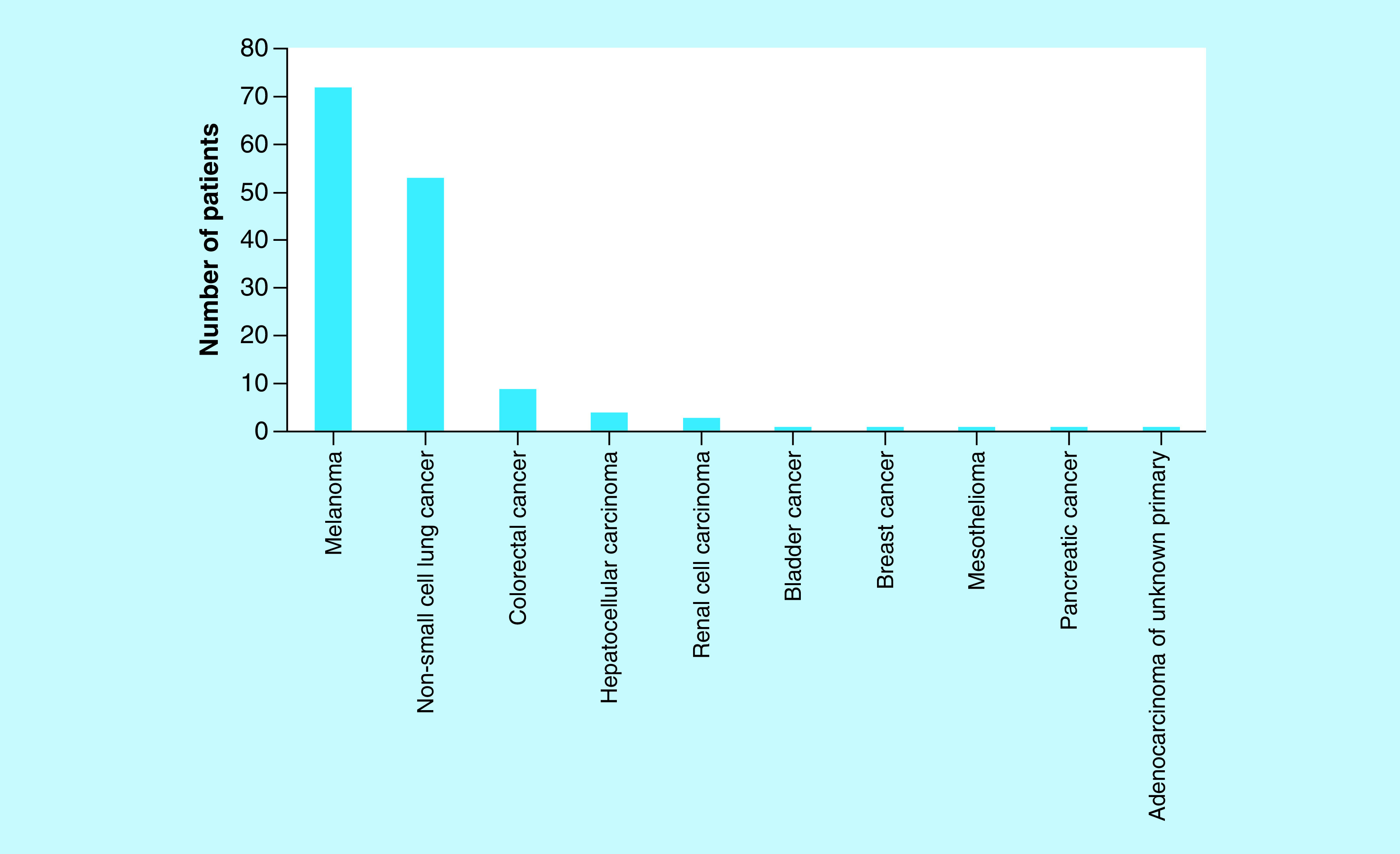
Cancer types. The majority of patients had melanoma or non-small-cell lung cancer, but there were a variety of other cancers treated with immune checkpoint inhibitors that were included.

The majority of patients had melanoma or non-small-cell lung cancer, but there was a variety of other cancers treated with immune checkpoint inhibitors that were included in the analysis (Figure 1). A total of 90% had stage IV disease, but a small proportion had earlier stage disease with treatment in the adjuvant setting. With regards to known poor prognostic factors, 12% of patients had brain metastases, and approximately half had a raised LDH at baseline.

**Figure 2.**
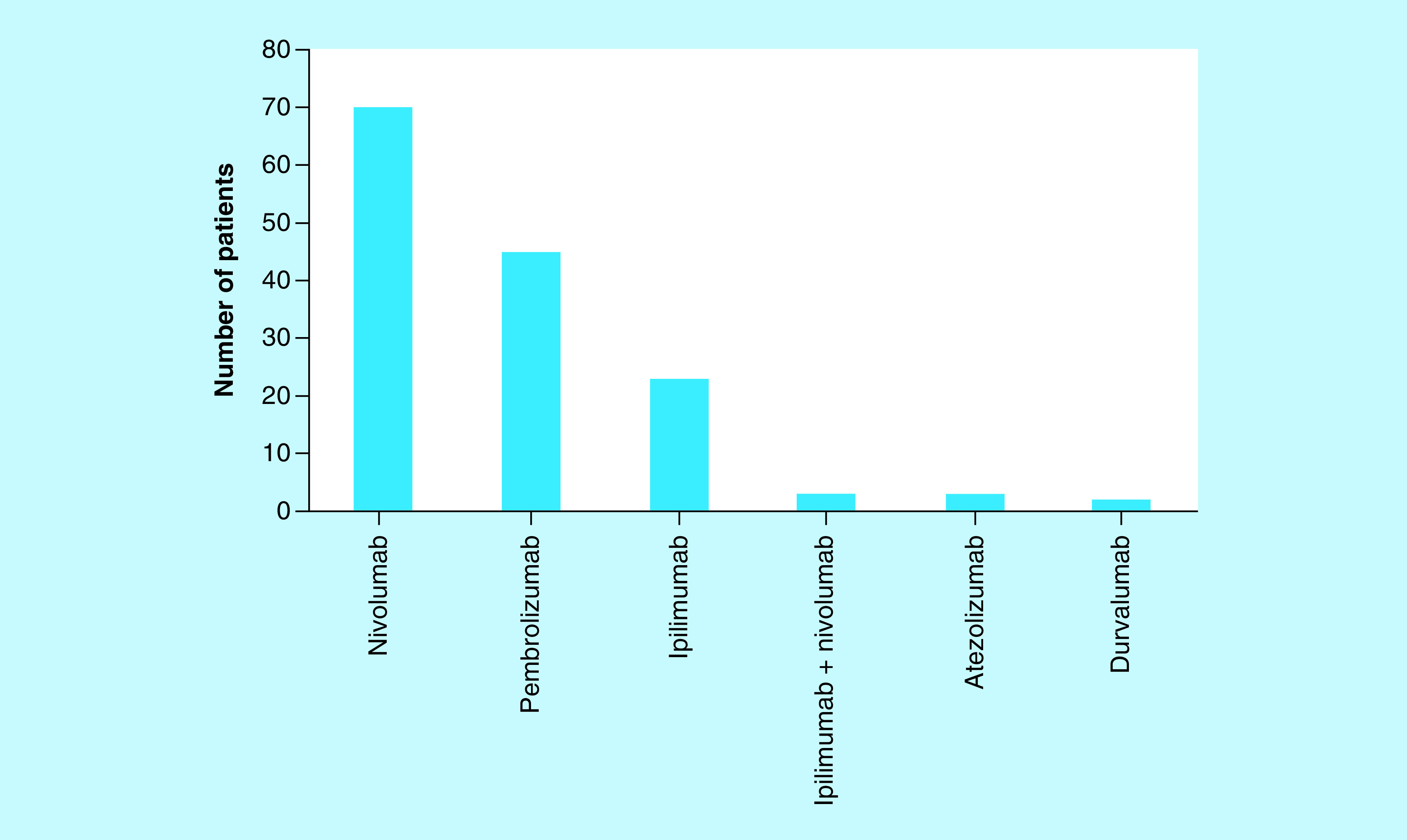
Immunotherapy treatment. Most patients received either pembrolizumab or nivolumab, but there were 24 patients who received ipilimumab and three patients who received combination ipilimumab plus nivolumab. A small number of patients were treated with the PD-L1 inhibitors atezolizumab or durvalumab.

The median number of immunotherapy cycles received per patient was 12 (range 1–84 cycles). The majority received either pembrolizumab or nivolumab, but there were 24 patients who received ipilimumab and three patients who received combination ipilimumab plus nivolumab (Figure 2). Five patients were treated with the PD-L1 inhibitors atezolizumab or durvalumab.

Regarding treatments prior to immunotherapy, 45% of patients had received radiotherapy. At the time of immunotherapy treatment commencement, 12 patients were receiving corticosteroids at a dose equivalent to prednisolone 10 mg daily or higher.

### Eosinophilia

The mean eosinophil count at baseline was 0.17 × 10^9^/l (range 0–1.31 × 10^9^/l). This rose to a mean eosinophil count of 0.24 × 10^9^/l at week 6 of immunotherapy treatment (range 0–1.81 × 10^9^/l) (p = 0.007).

Out of the 146 included patients, 22% developed eosinophilia while on immune checkpoint inhibitors. Eosinophilia was present at baseline in 3.4% of patients, rising to 8.9 and 17.8% of patients by cycle 2 and week 6 of treatment, respectively. A small number of patients developed eosinophilia after week 6 of treatment (4.1%).

### Treatment response

Data for 120 patients was available for the treatment response analysis. Disease control (complete response, partial response or stable disease) was seen in 65% of patients (Table 2).

**Table 2.** Immunotherapy treatment response.

Treatment response (120 patients included)	Number (% of total cohort)	Number with eosinophilia (% of column 2)
CR	7 (4.8)	1 (14.2)
PR	33 (22.6)	12 (36.3)
SD	39 (26.7)	9 (23.1)
PD	41 (28.1)	7 (17.1)

Most patients had a partial response or stable disease. Disease control (complete response, partial response or stable disease) was seen in 65% of patients. There was a significant association between the development of eosinophilia during treatment and achieving disease control (p = 0.009).

CR: Complete response; PD: Progressive disease; PR: Partial response; SD: Stable disease.

There was a significant association between the development of eosinophilia during treatment and achieving disease control (p = 0.009). With regards to objective response (complete or partial response), this was seen in 40 patients (33.3%). There was no significant association observed with emerging eosinophilia on treatment and objective response (p = 0.173). The correlation between the degree of eosinophilia and tumor response was not assessed in this study.

The binary logistic regression analysis determined that every 0.1 × 10^9^/l increase in the eosinophil count on treatment was associated with a 28% relative increase in the chance of achieving disease control (p = 0.034).

### Survival

**Figure 3.**
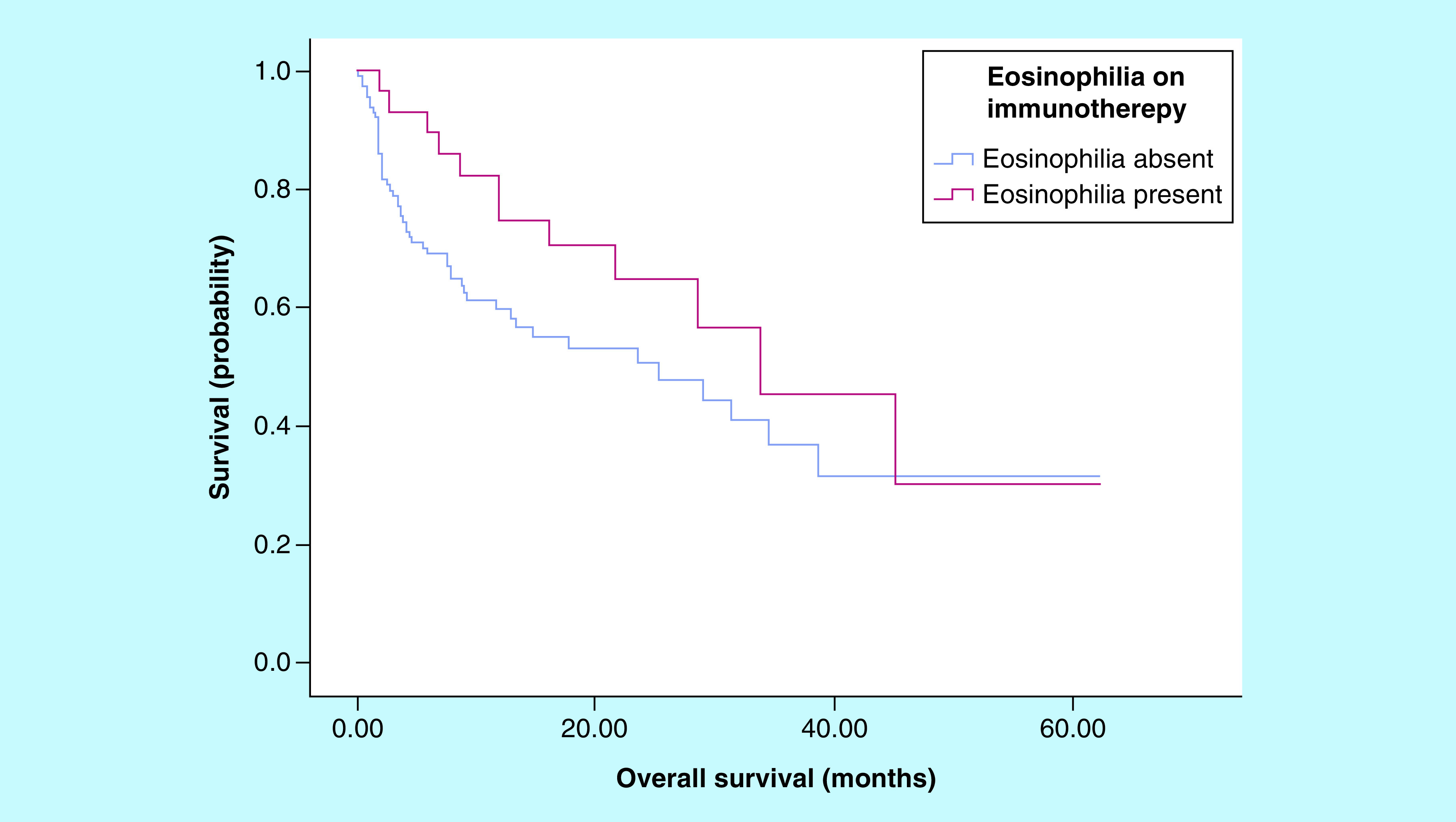
Kaplan–Meier curve for overall survival. The median overall survival was 25.32 months (95% CI 9.63–41.01 months) for those without eosinophilia, versus 33.83 months (95% CI 17.05–50.61 months) for those with eosinophilia on treatment. There was no significant association found between the development of eosinophilia on treatment and improved overall survival (p = 0.136).

Figure 3 demonstrates the Kaplan–Meier survival analysis for the total cohort. The median overall survival was 25.32 months (95% CI 9.63–41.01 months) for those without eosinophilia, versus 33.83 months (95% CI 17.05–50.61 months) for those with eosinophilia on treatment. Although there was separation of the curves seen, there was no significant association identified between the development of eosinophilia on treatment and improved overall survival (p = 0.136). The median overall survival of the 64 patients with melanoma was 45.11 months (95% CI 21.5–68.7 months), and for the 52 patients with non-small-cell lung cancer was 17.72 months (95% CI 4.36–31.08 months).

### Toxicity

Data for 142 patients was available for the treatment toxicity analysis. Immune related adverse events occurred in 47% of patients, with 10% experiencing severe (grade 3 or 4) toxicity (Table 3). The toxicities experienced were as expected for patients treated with immune checkpoint inhibitors, and there were no treatment-related deaths.

**Table 3.** Immunotherapy treatment toxicity.

Toxicity	Number (% of total cohort)	Number with eosinophilia (% of column 2)
Any grade	66 (46.6)	19 (28.7)
Grade 3 or 4	15 (10.5)	3 (20)

Immune related adverse events occurred in 47% of patients, with 10% experiencing severe (grade 3 or 4) toxicity. There was a significant association found between eosinophilia on treatment and the development of toxicity of any grade (p = 0.042), but not with severe toxicity.

There was a significant association observed between eosinophilia while on treatment and the development of toxicity of any grade (p = 0.042). With regards to severe toxicity, there was no association with eosinophilia (p = 1). Patients who developed eosinophilia while receiving treatment were not more likely to have particular toxicities when compared with those without eosinophilia. The effect of prednisolone and other treatments for toxicity on the eosinophil count was not assessed in this study.

## Discussion

Eosinophilia is defined as a peripheral blood absolute eosinophil count greater than 0.5 × 10^9^/l. Hypereosinophilia refers to moderate-to-severe eosinophilia (1.5 × 10^9^/l) on two separate occasions one month apart or pathologic confirmation of tissue hypereosinophilia [12]. There are a variety of conditions that can cause eosinophilia, including allergic reactions, infections and other immunologic processes such as vasculitis. Rarely, hypereosinophilia can be seen in malignant conditions including adenocarcinoma, Hodgkin lymphoma and T-cell lymphoma.

Despite the function of eosinophilia being well-defined in allergic and other immunologic conditions, the association with cancer and eosinophils remains unknown. In particular, the exact mechanism for why some patients develop eosinophilia on immunotherapy is not yet clear. Both peripheral and tumor-associated eosinophilia has been associated with improved prognosis in multiple cancers [9,13–15]. However, eosinophilia could also merely be a surrogate marker for a certain type of microenvironment caused by immune checkpoint inhibition.

Eosinophils can invade the tumor microenvironment and may enhance antitumor responses via degranulation with direct cytotoxic effects on cancer cells. This appears to occur in higher numbers in some patients, perhaps because these people generate more eosinophils due to their activated immune system and Th2 production of IL5. Furthermore, cytokines released from eosinophil granules are involved in the activation of dendritic cells, recruitment of T cells and alteration of the tumor microenvironment vasculature. At last, eosinophils may play a role in the enhancement of tumor surveillance [10,11,16]. There is evidence that eosinophilia is also linked to the development of immune related adverse events, alongside the mechanisms by which eosinophils affect the tumor microenvironment described above, may also play a role in how these toxicities develop. Then again, eosinophilia may merely be a consequence of these toxicities. It would be of great utility in our opinion to understand these processes in greater detail.

In our cohort, we determined the incidence of eosinophilia to be 22%. Patients receiving immunotherapy who developed eosinophilia were more likely to achieve disease control, but also develop treatment toxicity. Although there was a trend toward improved survival noted between 20 and 40 months, there was no statistically significant association between eosinophilia and improved overall survival.

These results support the role for eosinophilia as a prognostic and potentially predictive biomarker for patients receiving immunotherapy, which should be further explored in prospective studies. Given that the eosinophil count is part of the routine complete blood examination performed for all patients receiving immunotherapy, data regarding eosinophilia should be readily available. With the correlation observed with treatment response and toxicity, consideration should be given to reporting eosinophilia in prospective studies of immunotherapy agents. The majority of patients in our cohort had melanoma and non-small-cell lung cancer, and the majority of the current studies available regarding eosinophilia on immune checkpoint inhibitors are in the setting of these cancers. In relation to day-to-day clinical practice, our results suggest that in this cohort of patients who develop eosinophilia, we may be able to predict a higher chance of a treatment response or stable disease. With regards to toxicity, perhaps if a clinician sees eosinophilia on the blood film, they should have a heightened concern that toxicity may develop. In this regard, emerging eosinophilia while receiving treatment may act as a ‘red flag’ for the clinician to monitor the patient more closely.

Given the association identified between a rise in eosinophil count on treatment and achieving disease control, it may be useful to develop a grading system for eosinophilia, similar to the grading system for other immune related adverse events. However, at this point, there is a lack of prospective data regarding the clinical consequences of eosinophilia and hypereosinophilia in patients receiving immune checkpoint inhibitors. The current Common Terminology Criteria for Adverse Events grading system for eosinophilia is somewhat vague, and could be better characterized to report eosinophilia, particularly in clinical trials, such that this marker can be further explored. There may be patients in whom improved outcomes or worse toxicity can be predicted earlier.

For day-to-day clinical use, a proposed protocol for the assessment of patients who develop eosinophilia while on immune checkpoint inhibitors is demonstrated in Box 1.

Box 1. **Proposed protocol for the assessment of patients who develop eosinophilia on immune checkpoint inhibitor therapy.**

- Consider other causes of eosinophilia (for example, parasitic or fungal infection, allergy/atopy, drug reactions etc.).
- Examine the trend in eosinophil count for the period during which the patient has been on an immune checkpoint inhibitor.
- At the time of next review, re-check the eosinophil count and assess the following:
  - Symptoms or signs of treatment response
  - Symptoms or signs of treatment toxicity
- If 12 or more weeks since last staging scan, consider repeating scan to assess for disease control.
- Depending on severity of eosinophilia and clinical features found above, consider earlier repeat review.

Strengths of this study are that it provides real-world data on the incidence of eosinophilia in a patient population receiving immunotherapy, and the potential use of this as a biomarker. In addition, this was a heterogeneous patient population, which overall adds to the body of literature available regarding eosinophilia for patients with multiple cancer types and various immunotherapy agents.

There are a number of limitations of the study, in particular that it is a retrospective, single center study. The small patient population also led to a lack of power to detect particular associations, such as regarding objective response or severe toxicity. We feel there would be a benefit in performing a prospective trial to better define the patients who develop eosinophilia and correlate this to toxicity, tumor response and disease specific survival. Confirming whether response and toxicity are mutually exclusive would also be important. Grading eosinophilia may also correlate with the degree of findings we hypothesize. At last, it would also be interesting to prospectively explore whether peripheral blood eosinophilia is associated with the presence of eosinophils in the tumor microenvironment.

## Conclusion & future perspective

This study demonstrates a significant association between eosinophilia and achieving disease control for patients on immune checkpoint inhibitors. Additionally, patients experiencing treatment toxicity were more likely to have eosinophilia on treatment. Eosinophilia is a simple and potentially useful biomarker and further prospective randomised studies are required to assess its clinical utility for patients on immunotherapy.

**Executive summary**

- Biomarkers to predict response and toxicity to cancer immunotherapy have far-reaching implications.
- In this retrospective study, patients who developed eosinophilia on treatment were more likely to achieve disease control.
- A rise in eosinophil count on treatment was associated with an increased chance of achieving disease control.
- Treatment toxicity was more likely in patients who developed eosinophilia on treatment.
- Eosinophilia is a simple and potential useful biomarker, worthy of further prospective study.
